# Rough and Porous Micropebbles of CeCu_2_Si_2_ for Energy Storage Applications

**DOI:** 10.3390/ma16227182

**Published:** 2023-11-16

**Authors:** Davide Scarpa, Claudia Cirillo, Christopher Luciano, Angela Nigro, Renata Adami, Carla Cirillo, Carmine Attanasio, Mariagrazia Iuliano, Eleonora Ponticorvo, Maria Sarno

**Affiliations:** 1 Department of Physics “E.R. Caianiello”, University of Salerno, Via Giovanni Paolo II, 132, 84084 Fisciano, Italy; clcirillo@unisa.it (C.C.); chluciano@unisa.it (C.L.); anigro@unisa.it (A.N.); radami@unisa.it (R.A.); cattanasio@unisa.it (C.A.); maiuliano@unisa.it (M.I.); eponticorvo@unisa.it (E.P.); msarno@unisa.it (M.S.); 2 NANO_MATES Research Centre, University of Salerno, Via Giovanni Paolo II, 132, 84084 Fisciano, Italy; 3 CNR-SPIN, c/o University of Salerno, Via Giovanni Paolo II, 132, 84084 Fisciano, Italy; carla.cirillo@spin.cnr.it

**Keywords:** lanthanide elements, CeCu_2_Si_2_, supercapacitor, high capacitance, high stability

## Abstract

Supercapacitors have attracted considerable attention due to their advantages, including being lightweight and having rapid charge–discharge, a good rate capability, and high cyclic stability. Electrodes are one of the most important factors influencing the performance of supercapacitors. Herein, a three-dimensional network of rough and porous micropebbles of CeCu_2_Si_2_ has been prepared using a one-step procedure and tested for the first time as a supercapacitor electrode. The synthesized material was extensively characterized in a three-electrode configuration using different electrochemical techniques, such as cyclic voltammetry (CV), galvanostatic charge and discharge (GCD) tests, and electrochemical impedance spectroscopy (EIS). CeCu_2_Si_2_ shows rather high mass-capacitance values: 278 F/g at 1 A/g and 295 F/g at 10 mV/s. Moreover, the material exhibits remarkable long-term stability: 98% of the initial capacitance was retained after 20,000 cycles at 10 A/g and the Coulombic efficiency remains equal to 100% at the end of the cycles.

## 1. Introduction

In the present day, the escalating global demand for electrical energy has sparked a significant demand for energy storage solutions capable of delivering top-notch performance. Indeed, this technology plays a crucial role in numerous stand-alone applications. On the other hand, within the context of distributed renewable energy systems, energy storage holds a pivotal position, not only serving stand-alone systems but also facilitating the integration of these renewable sources into the electrical grid. In this context, energy storage takes on a central role in the effort to combine forthcoming sustainable energy supply with the established norms of technical services and products. Numerous energy storage technologies have been developed, including electrochemical energy storage, fluid-based storage, mechanical systems, and electromagnetic solutions. These diverse energy storage methods coexist due to their unique characteristics, which makes them suitable for various specific applications [1].

In the field of electrochemical energy storage (EES), supercapacitors have drawn increasing attention in addressing the emerging energy demand, primarily because of their plethora of advantages over conventional capacitors and batteries. These advantages encompass superior power densities, exceptional long-cycle lifespans, fast charge–discharge characteristics, adaptability to a wide range of temperatures, as well as improved eco-friendliness [2,3,4].

Supercapacitors, as an energy storage solution, can be conveniently classified into three primary categories: electrical double-layer capacitors (EDLCs), redox capacitors, and pseudocapacitors. Among these, EDLCs operate on a distinct energy storage principle based on the formation of a dual layer of ions in close proximity to the electrode surfaces, a phenomenon commonly referred to as the Helmholtz electrical double layer. Owing to this specific mechanism, electrical double-layer capacitors can deliver exceptional power densities, relying on the rapid kinetics involved in physical ion adsorption and desorption. However, it is important to note that EDLCs typically exhibit lower capacitances and energy density when compared to the other categories. In contrast, redox capacitors are based on an alternative energy storage mechanism that revolves around redox reactions occurring at the interfaces between the electrodes and the electrolyte. These redox reactions can also occur within the bulk of the electrode material, endowing redox capacitors with higher capacitance compared to their EDLC counterparts. Nevertheless, the charge–discharge process in redox capacitors, which results from redox reactions, is notably slower than the physical mechanism of EDLCs. Consequently, redox capacitors are associated with reduced power densities along with higher capacitances. Ultimately, pseudocapacitors emerge as a category that bridges the gap between the previously discussed groups. The charge storage mechanism in pseudocapacitors is characterized by rapid redox reactions at the electrode–electrolyte interface so that the electrochemical signature of a pseudocapacitor electrode is close to the one of EDLSCs. This unique mechanism, often referred to as “pseudocapacitance”, confers upon pseudocapacitors the dual benefit of ensuring elevated power and energy densities [5,6]. 

On the other hand, lanthanide (Ln) elements have been extensively investigated across various application domains, including the display industry, magnets, bio-industry, pyro-processing technology in nuclear power plants, and energy/environment fields [7,8,9,10,11,12,13,14,15,16]. Indeed, these elements exhibit unique physicochemical properties due to their unpaired 4f electronic configurations. The closely matched energy levels of the 4f and 5d orbitals enable efficient electron transfer between these orbitals, which in turn promotes redox processes. Additionally, Ln elements can effectively store a higher number of charges due to their multiple oxidation states and the capacity to undergo reversible redox reactions. These features can contribute to improving charge–discharge rates as well as capacitance, making these materials suitable as supercapacitor electrodes [17,18]. Among alloy systems including Ln elements, CeCu_2_Si_2_ has drawn substantial attention due to its distinctive superconducting properties, with research extending back to the previous century. In 1979, indeed, a groundbreaking discovery arose within the domain of condensed matter physics since superconductivity was observed for the first time in the magnetic material CeCu_2_Si_2_. This marked a significant milestone as it introduced the scientific community to an entirely new category of superconductors, often referred to as unconventional non-phonon-mediated superconductors. Notably, in these unconventional superconductors, there is a prevailing suspicion that the mechanism responsible for the formation of Cooper pairs, which is responsible for superconductivity, is intrinsically connected to magnetism. In the context of intermetallic compounds, the electronic degrees of freedom, crucial for facilitating superconductivity, exhibit a direct and intricate relationship with the magnetic moments originating from the partially filled f shell of the atoms involved [19].

On the other hand, as a result of the collaborative contribution of various constituent elements, which can provide interesting features and fast redox reactions due to a synergistic effect of electronic conduction and enhanced oxidation states, CeCu_2_Si_2_ material emerges as an intriguing and innovative prospect for exploration as a supercapacitor electrode offering promising possibilities in this regard.

In particular, silicon (Si) has recently drawn increasing attention in the energy storage field due to its low working potential and high theoretical capacity (around 4200 mA h g^−1^). Moreover, silicon is the second most abundant element on Earth, which means there is a stable and sustainable supply. This, combined with its low cost and environmentally safe properties, places silicon in a favorable position as a viable choice for supercapacitor electrode materials [20,21,22]. To enhance the electrical conductivity and the specific capacitance of the electrode, the combination of several elements with Si to form a ternary material can be an effective method. On the other hand, copper (Cu) is one of the most suitable and effective candidates because of its low cost, good electrical conductivity, and environmentally friendly nature [23,24]. Additionally, density functional theory (DFT) calculations proved that the introduction of Cu atoms into another element’s lattice could enhance the electron transport rate, therefore increasing the overall electrochemical activity, as a consequence of the introduction of new energy levels near the Fermi level, originated from the 3D orbitals of Cu [25,26]. Cerium (Ce), another element considered for integration with silicon, possesses high electrical conductivity [27,28] and a rich energy-level structure [29]. These qualities enhance the inherent activity of the electrode material and improve its electrochemical performance. Indeed, Ce exhibits enhanced pseudocapacitive features owing to the fast oxidation and reduction of the Ce^3+^/Ce^4+^ ions [30]. Overall, combining Cu and Ce metals in an electrode enables one to obtain an increased number of active sites and several metal ion valence states, resulting in an additional synergistic effect of electronic conduction [31].

Furthermore, apart from composition, morphology stands as another critical element that demands careful consideration when aiming to design an efficient supercapacitor electrode. Indeed, porosity [32] and rough surfaces [33] increase wettability and the available interface area between electrodes and electrolytes.

In the following study, a rough-surface and porous ternary CeCu_2_Si_2-_based material, prepared via a one-step synthetic procedure, has been tested for the first time as a supercapacitor electrode. The synthesized material was first characterized from a morphological point of view. Eventually, its electrochemical performance was investigated via cyclic voltammetry, galvanostatic charge–discharge tests, and electrochemical impedance spectroscopy carried out in a three-electrode-setup-based electrolytic cell filled with a H_2_SO_4_ aqueous solution.

## 2. Materials and Methods

### 2.1. CeCu_2_Si_2_ Preparation

CeCu_2_Si_2_ was synthesized starting from elements with high purities (>99.9%, Sigma-Aldrich, St. Louis, MO, USA) in a molten flux of the different elements. The reaction mixture was obtained by loading the Ce, Cu, and Si elements in the optimized ratio of 1:13:11.44 into a 5 mL alumina crucible, which was then covered by a second inverted crucible filled with silica wool. Such a system was sealed under vacuum in a quartz ampoule and heated to 800 °C at 50 °C/h. Afterward, it was maintained at 800 °C for 7 h, further heated to 1200 °C at 50 °C/h, kept at 1200 °C for 12 h, and eventually cooled down at 2 °C/h to 900 °C. At this temperature, the ampoule was quickly removed from the furnace and inverted; this way, the silica wool acted as a sieve for separating most of the liquid flux from the solid material. Afterward, the ampoule was inserted into a centrifuge with the purpose of further separating the remaining liquid flux and recovering the powder. 

### 2.2. Characterization

First of all, the samples were characterized by size, morphology, composition, porosity, and structure. For these purposes, the electron microscope, which is one of the most powerful analytical tools available for characterizing microscopic particulate material, allowing the examination of particle morphology and surface details, was used. In particular, Scanning Electron Microscopy (SEM) used in tandem with Energy-Dispersive X-ray Microanalysis (EDX) is a combination of instrumentation that is capable of providing detailed information on the size and composition at individual particle levels, with the strength of gathering data in a time-efficient manner. SEM images were obtained with a TESCAN-VEGA LMH (230 V) microscope (TESCAN, Brno, Czech Republic), equipped with an EDX probe. The X-ray diffraction analysis (XRD) of the sample was obtained through a Bruker D8 X-ray diffractometer (Bruker Corporation, Billerica, MA, USA), with monochromatic CuKa (λ = 0.1542 nm) radiation, operating at 40 kV and 30 mA. The diffraction pattern was collected at 25 °C and over an angular range of 10° to 40°. The BET method was used to measure the physisorption of nitrogen, passing between particles and into pores, cracks, and surface roughness, to derive the value of the “surface area”. The N_2_ adsorption–desorption isotherm was obtained at −196 °C. In particular, the surface area was evaluated using Brunauer–Emmett–Teller (BET) with a Sorptometer 1042 instrument (Costech International, Milan, Italy). Before the analysis, the sample was pre-treated at 150 °C for 60 min under a He flow. The Barrett–Joyner–Halenda (BJH) method was used to determine the pore-size distribution of the material.

Cyclic voltammetry, galvanostatic charge and discharge tests, and electrochemical impedance spectroscopy experiments were carried out using an Autolab PGSTAT302N potentiostat (Metrohm, Herisau, Switzerland) in a three-electrode-setup-based electrolytic cell filled with a 1 M H_2_SO_4_ solution. Saturated calomel, graphite, and a loadable glassy carbon ring (3 mm diameter) were adopted, respectively, as reference, counter, and working electrodes [34].

In preparation for the electrochemical tests, 8 mg of the material was mixed into 160 μL of a 5 wt% Nafion solution, 900 μL of 2-propanol, and 100 μL of water to create a stable and conductive homogenous ink. This ink was then partially drop-casted onto the working electrode and dried under an ambient environment to attain a uniform catalyst film with a mass loading of about 2 mg/cm^2^. 

The EIS spectra were taken at open circuit voltage (OCV) using a 5 mV amplitude at frequencies ranging from 200 kHz to 10 MHz.

The mass capacitance (Csp, F/g) of the electrode has been calculated from galvanostatic charge–discharge curves according to the following equation:

(1) $$Csp=\frac{i\cdot ∆t}{m\cdot ∆V}$$

where *i* is the GCD current, ∆*t* is the discharging time, ∆*V* is the potential window, and *m* is the loading mass of the electrode material.

Moreover, capacitance retention at the *n*th cycle has been evaluated according to the following equation:

(2) $$Capacitance\;retention\;(n\;cycle,\%)=\frac{Csp\;(n\;cycle)}{Csp}\cdot 100$$

where *Csp* (*n cycle*) is the mass capacitance calculated at the *n*th cycle (with *n* = 2, 3… 20,000), while *Csp* is the mass capacitance calculated at the 1st cycle.

Eventually, Coulombic efficiency has been evaluated according to the following equation:

(3) $$Coulombic\;efficiency\;(n\;cycle,\;\%)=\frac{Csp,discharge\;(n\;cycle)}{Csp,\;\;charge\;(n\;cycle)}\cdot 100$$

where *Csp, discharge* (*n cycle*) is defined as the measured discharge mass capacity at the *n*th cycle, whereas *Csp, charge* (*n cycle*) is the measured charge mass capacity at the same *n*th cycle, calculated from (1) considering ∆*t* as the charging time [35,36,37].

Energy density (*E*, Wh/kg) and power density (*P*, W/kg) have been evaluated according to the following formulas:

(4) $$E=\frac{1}{2}Csp\cdot \;{∆V}^{2}\frac{1000}{3600\;}$$

(5) $$P=\frac{E}{∆t}\;$$

where *Csp* is the mass capacitance calculated in (1), ∆*t* is the discharging time, and ∆*V* is the potential window from the galvanostatic charge–discharge curves.

## 3. Results and Discussion

### 3.1. Morphological Characterization

**Figure 1:**
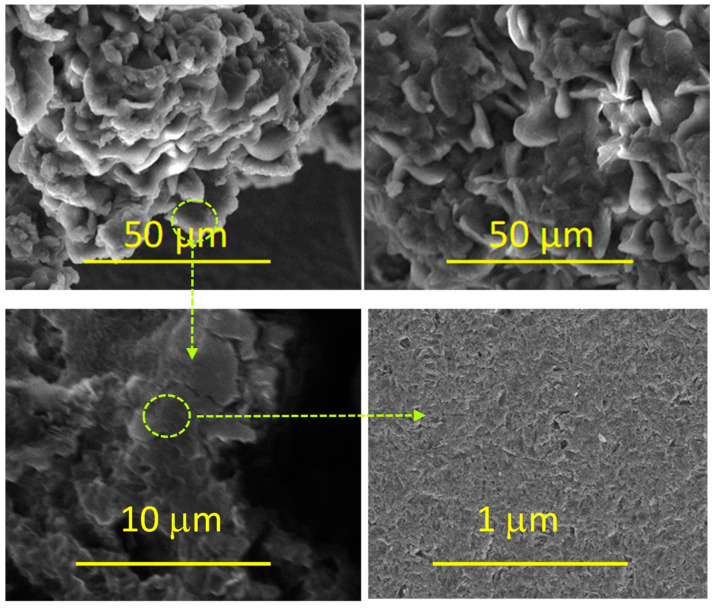
SEM images of CeCu_2_Si_2_.

**Figure 2:**
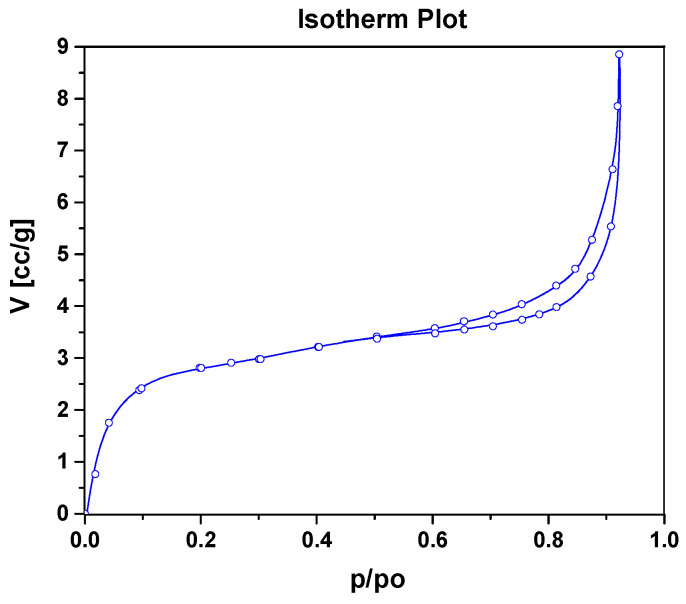
Nitrogen adsorption isotherms at 77 K of CeCu_2_Si_2_.

The morphological features of the CeCu_2_Si_2_ were investigated through SEM analysis. The results of SEM analysis on hard surfaces are reported in Figure 1. As can be seen from Figure 1, the material is in the form of a three-dimensional network of micropebbles, strictly connected to each other, showing a rough and porous surface. Indeed, the BET surface area for CeCu_2_Si_2_, obtained via the nitrogen adsorption–desorption isotherm (Figure 2) is equal to 12 m^2^/g, and the total pore volume and the micropore volume are 0.14 cm^3^/g and 0.001 cm^3^/g, respectively. The multi-modal pore size distribution (BJH pore distribution) is centered at 26.5 nm (inter-particle surface porosity) and 16 nm (intraparticle surface porosity).

**Figure 3:**
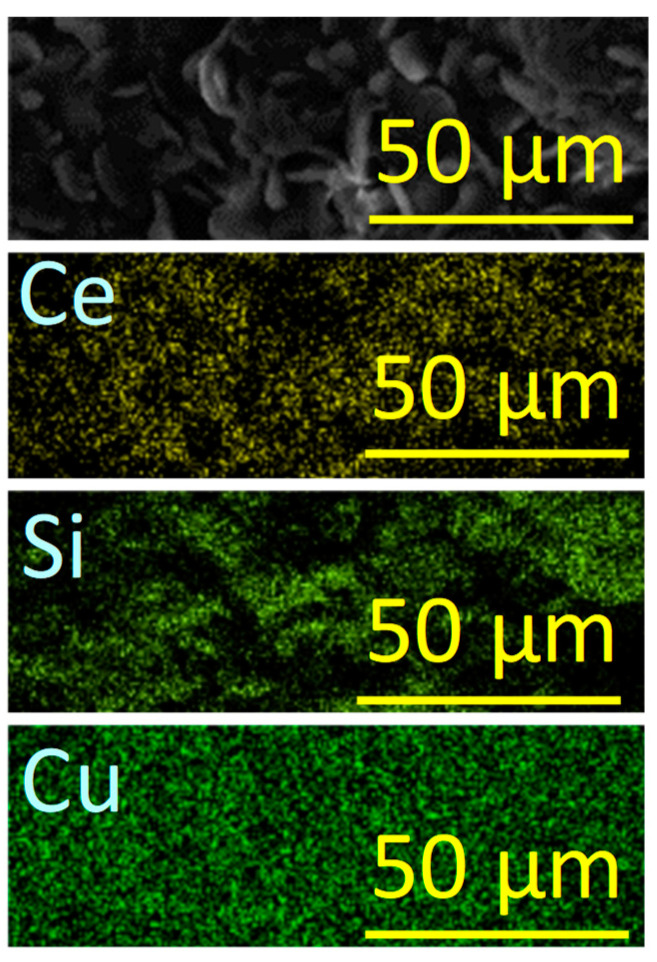
EDX analysis on CeCu_2_Si_2_ with an SEM image and the corresponding EDX maps for Ce, Si, and Cu.

Moreover, EDX maps were collected to verify the homogeneity and chemical composition of the prepared sample. As displayed in Figure 3, all constituent elements (Ce, Si, and Cu) are distributed homogeneously within the sample. The real chemical formula CeCu_2_Si_2_ was also confirmed.

**Figure 4:**
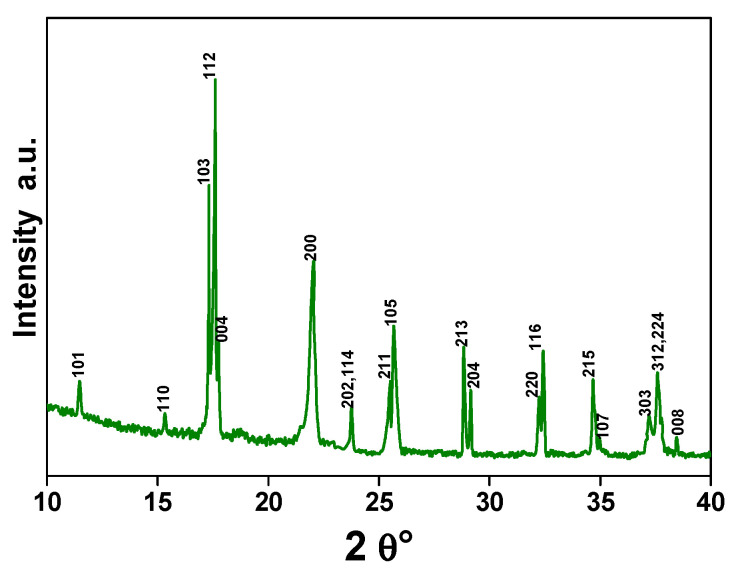
X-ray diffraction pattern of CeCu_2_Si_2_.

The X-ray diffraction pattern in Figure 4, showing the typical peaks of the tetragonal crystal structure of CeCu_2_Si_2_, confirms the nature of the sample [38]. The intensities of the diffraction lines are in agreement with what would be expected for a randomly oriented polycrystalline material. In particular, the peaks indexes can be referred to as the ones of the ThCr_2_Si_2_ structure type, with the Ce, Cu, and Si atoms located, respectively, on the 2a (0,0,0), 4d (0,0.5,0.25), and 4e (0,0,z) Wyckoff sites of the space group I4/mmm [39,40]. 

### 3.2. Electrochemical Characterization

**Figure 5:**
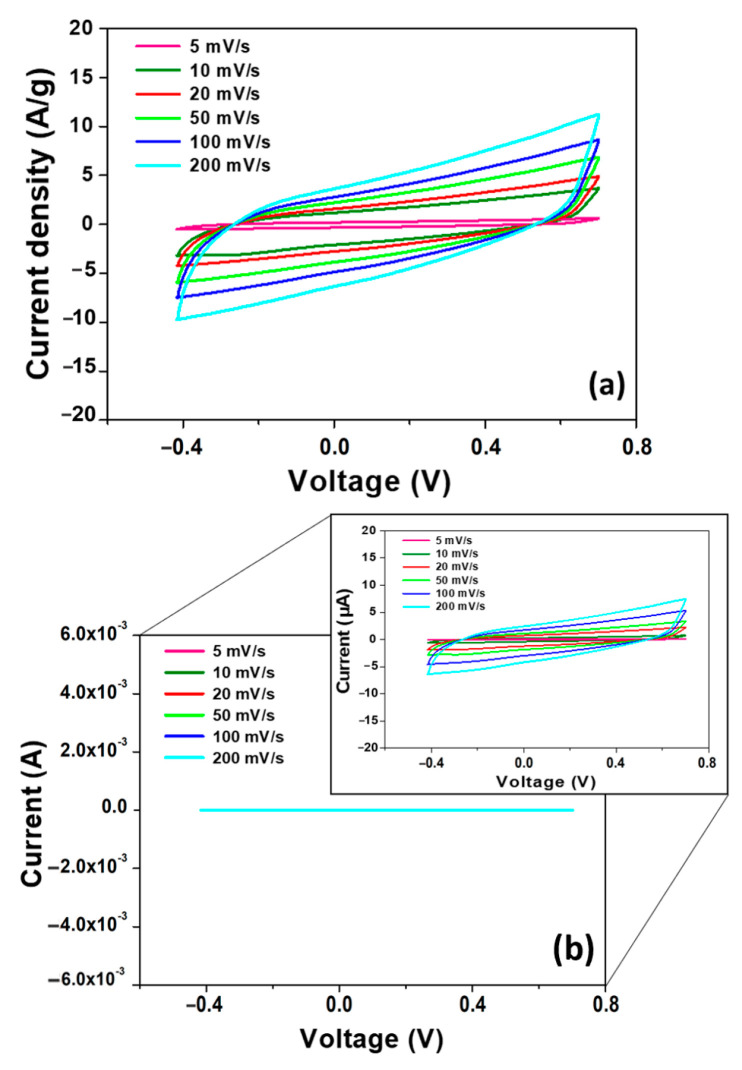
(**a**) CV curves of CeCu_2_Si_2_ at different scan rates. (**b**) CV curves of the bare glassy carbon electrode at different scan rates. In the inset, a magnification of the same graph, with the current values expressed in µA, is reported.

The capacitive behavior of the CeCu_2_Si_2_ electrode was first investigated in a three-electrode-setup-based electrolytic cell filled with a 1 M H_2_SO_4_ aqueous electrolyte solution via cyclic voltammetry. The electrochemical curves were recorded in the potential range −0.4÷0.7 V at several scan rates (5, 10, 20, 50, 100 and 200 mV/s). As can be seen in Figure 5a, the material shows an approximately quasi rectangular current–voltage response without detectable redox peaks, suggesting pseudocapacitive behavior. The first definition of pseudocapacitance was proposed by B.E. Conway in [41]. In particular, pseudocapacitance was suggested to arise at the electrode surfaces where materials undergo fast and reversible redox reactions, which is reflected in an electrochemical signature of (quasi)-rectangular CV curves similar to EDLC. The absence of visible peaks here can be ascribed to fast surface redox reactions, suggesting that the charge transport is taking place at high rates. The lack, even at lower scan rates, may be representative of the equitable contribution to the capacitance of both the electrostatic and the redox storage mechanisms [42,43,44,45]. On the other hand, regarding redox pseudocapacitance, a contribution can be expected to be made by synergy between the Ce and Cu elements, consisting of a higher tendency to reduction and oxidation, respectively, of the Ce^4+^/Ce^3+^ and Cu^+^/Cu^2+^ redox couples as well as an improved charge transfer between them [31], as will be further explained in Section 3.3. Moreover, the CV curves of the bare glassy carbon electrode at the same current densities (see Figure 5b), exhibiting currents of only a few µA, enabled us to rule out a significant contribution of the bare electrode to the material capacitance.

**Figure 6:**
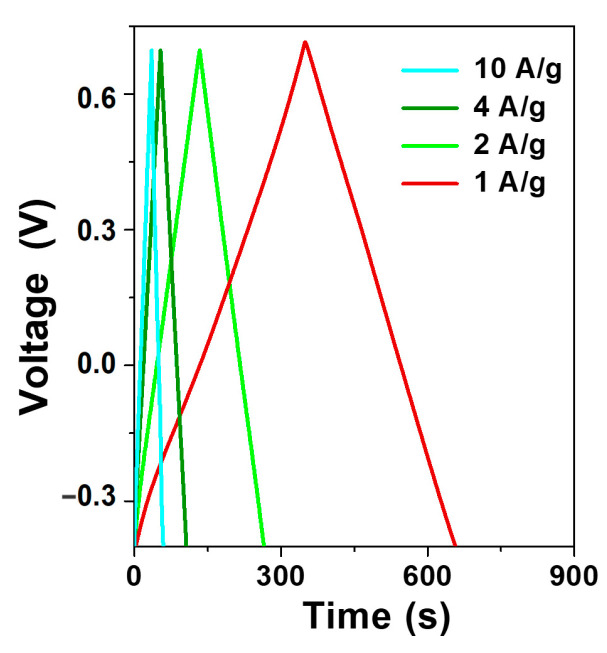
Galvanostatic charge–discharge curves of CeCu_2_Si_2_ at different current densities.

GCD tests were also performed in the current density range of 1 ÷ 10 A/g, and the results are reported in Figure 6; they show quasi-linear charge and discharge curves. There is no obvious IR drop even at the high current density of 10 A/g, probably due to the high electrode conductivity, enhanced by the intimate connection between the elements in the material.

**Figure 7:**
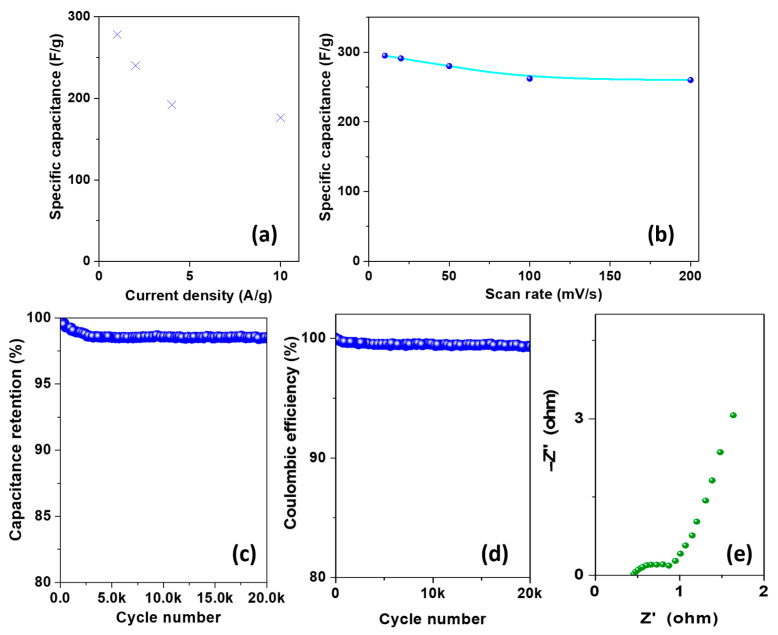
(**a**) Mass capacitance vs. current density, (**b**) mass capacitances of CeCu_2_Si_2_ at different scan rates, (**c**) capacitance retention of CeCu_2_Si_2_ as a function of cycle number, (**d**) Coulombic efficiency as a function of cycle number, and (**e**) Nyquist plot for CeCu_2_Si_2_.

From the galvanostatic discharge curve at 1 A/g, a mass-capacitance of 278 F/g was calculated according to (1). The mass capacitance, as expected, decreases at increasing current density, due to the reduced utilization of active material caused by the shorter time taken for the electrolyte ions to diffuse into the electrode channels. However, it still remains high (~180 F/g) at the high discharge current density of 10 A/g (see Figure 7a), with a retention of about 63% within this current range. Moreover, the sample exhibits a capacitance retention of about 89%, (see Figure 7b), with a mass capacitance of 295 F/g at a scan rate of 10 mV/s decreasing to only 262 F/g at 100 mV/s. Even in this case, the mass capacitance value steadily decreases with the increase in the scan rate since the electrolyte ions have less and less time for diffusion into the material as the scan rate increases. At a higher scan rate, a faster redox reaction happens, which reduces the electrolyte ion diffusion into the active material and consequently lowers the capacitance [46]. To further investigate the electrochemical processes, the relationship between current density and scan rate was investigated. Typically, the above-mentioned relationship is commonly expressed in the following manner:

(6) $$i=av^{b}$$

where *i* is the current density, *v* is the scan rate, and *a* and *b* are appropriate values. In this specific case, the value of b is 0.72, suggesting that the electrochemical process is not completely unconditioned by diffusion paths [47].

On the other hand, even when the material power density, calculated according to (5), reaches 47 kW·kg^−1^, its energy density, obtained according to (4), maintains a value of 72 Wh·kg^−1^.

Furthermore, the sample shows high durability in terms of capacitance retention, evaluated according to (2): in 1 M H_2_SO_4_ at a high current density of 10 A/g, the sample retains 98% of the initial capacitance over 20,000 cycles (Figure 7c). The Coulombic efficiency of the sample, which measures the electron transfer efficiency during the charge–discharge process, was also calculated according to (3), being nearly 100%, which can be attributed to the high charge–discharge rates of the material due to Ce and Cu, and it maintains its value, which is almost unchanged until the 20,000th cycle (Figure 7d). Eventually, electrochemical impedance spectroscopy was carried out to determine the resistivity, as well as the charge-transfer and ion diffusion behavior of the CeCu_2_Si_2_ material. Figure 7e illustrates the Nyquist plot exhibiting a semi-circle in the high-frequency region and a quasi-vertical line feature at a lower frequency related to the pseudo-capacitive contribution. In the middle-frequency region, an initial inclined portion of the curve (about 45°) can be attributed to the Warburg impedance, related to the resistance to the ion diffusion/transport from the electrolyte to the electrode [48]. In the high-frequency region, the intercept on the Z′ axis indicates the equivalent series resistance (ESR), i.e., the combination of the internal resistance of the electrode, the ohmic resistance of the electrolyte, as well as the contact resistance between the current collector and electrode. For the CeCu_2_Si_2_ material, an ESR of 0.42 Ω has been calculated, which indicates a rather small internal resistance. The nature of the material, its wettability ensured by the rough surface structure, as well as the path for ion diffusion due to mesopores, might be responsible for the interesting results, especially the non-trivial stability observed for the reported non-nanometric size material. These results encourage the use of this non-precious and cheaper material, non-conventionally considered and unexplored for supercapacitor application.

### 3.3. Comparison with Literature and Discussion

In Table 1, the capacitive performance of CeCu_2_Si_2_ is compared with the ones of some of the best-performing Cu, Ce, and/or Si-containing electrode materials for supercapacitor applications in the literature.

From the comparison, it is evident that the obtained material ranks among the best-performing ones, even when compared with finer-size materials and materials produced with less scalable processes. The CeCu_2_Si_2_ electrode exhibits a rather high specific capacitance at low current density, and, above all, it retains capacitance for a very high number of cycles. This can be attributed to several factors: the increase in the overall electrical conductivity due to the intimate electronic connection among the components; the rough surface and mesoporosity which guarantee both wettability with the electrolytic solution and ion diffusion path within the particles; very small internal resistance to charge transfer, which further extends the electrode durability.

Regarding Ce and Cu, they can exhibit enhanced pseudocapacitive features, owing to the fast reduction and oxidation, respectively, of the Ce^4+^/Ce^3+^ and the Cu^+^/Cu^2+^ redox couples. In particular [31], the synergistic effect between these two elements could have an important influence on the reaction mechanism. Indeed, having different standard reduction potentials (E° = 1.44 V for Ce^4+^/Ce^3+^; E° = 0.15 V for Cu^+^/Cu^2+^), Ce^4+^ is a stronger oxidant (7), whereas Cu^2+^ is a stronger reductant (9), with a contextual reduction of Ce^3+^ to Ce^4+^ and oxidation of Cu^2+^ to Cu, enabling fast charge transfer (8).

(7) $${Ce}^{4+}+e^{-}↔{Ce}^{3+}$$

(8) $${Ce}^{3+}+{Cu}^{2+}↔{Ce}^{4+}+{Cu}^{+}$$

(9) $${Cu}^{+}↔{Cu}^{2+}+e^{-}$$

## 4. Conclusions

In summary, rough-surface and porous-structure-based micropebbles of CeCu_2_Si_2_, a material typically known for its unique superconducting properties, have been successfully prepared with the aim of a simple one-step synthesis process and tested for the first time as a supercapacitor electrode. The X-ray diffraction analysis, which revealed the characteristic peaks associated with the tetragonal crystal structure of CeCu_2_Si_2_, confirmed the nature of the sample. Scanning electron microscope images at different magnifications, Brunauer–Emmett–Teller, and Barrett–Joyner–Halenda further elucidated the material morphology, indicating that CeCu_2_Si_2_ is in the form of a three-dimensional network of interconnected micropebbles, with a rough and mesoporous surface. On the other hand, the corresponding EDX maps confirmed that all the constituent elements (Ce, Si, and Cu) are distributed homogeneously within the material structure.

Afterward, the synthesized material was broadly characterized by different electrochemical techniques, and the capacitance performance was evaluated. Cyclic voltammetry and galvanostatic charge–discharge tests provided nearly rectangular and quasi-linear curves without plateaus, respectively, suggesting a pseudocapacitor behavior with an equitable contribution to the capacitance of both the redox and the electrostatic storage mechanisms. The good capacitive performance of CeCu_2_Si_2_ was confirmed by its rather high mass capacitance values (278 F/g at 1 A/g and 295 F/g at 10 mV/s), which remain high as both current density and scan rate increase. Furthermore, long-term cycling tests were performed, proving that the material retains its capacitance for a very high number of cycles. This behavior can be attributed to several factors: the increase in the overall electrical conductivity due to the intimate electronic connection among the components; the rough surface and mesoporosity which guarantee both wettability with the electrolytic solution and ion diffusion path within the particles; very small internal resistance to charge transfer which further extends the electrode durability. Moreover, the reported study demonstrates the potential to explore well-established materials in the domain of condensed matter physics, from both a chemical and structural perspective, and also in new emerging applications, such as in the EES field, where they can be found as really promising materials.

## Figures and Tables

**Table 1:** Supercapacitive performance of the CeCu_2_Si material and some of the best-performing Cu, Ce, and/or Si-containing electrode materials for supercapacitor applications in the literature.

Cu, Ce, Si-Containing Electrode Material (with Average Size)	CV Potential Window (V)	GCD Current Density (A/g)	Mass Capacitance (F/g_sample_)	Capacitance Retention	Ref.
Ce-doped NiO nanoflakes (27–30 nm)	0 ÷ 0.45 vs. Ag/AgCl	1	1775	retains about 93% after 2000 cycles at 5 A/g	[30]
CeO_2_ nanoparticles (14 nm)	0 ÷ 0.8 vs. Ag/AgCl	2	457	retains about 82% after 2000 cycles at 10 A/g	[27]
Ce-doped MnO_2_ nanorods (10–20 nm)	0 ÷ 0.8 vs. SCE	1	101.1	retains about 99.5% after 1000 cycles at 5 A/g	[49]
Cu-doped-MnO_2_ nanosheets	0 ÷ 1 vs. SCE	1	296	retains about 79% after 1000 cycles at 2 A/g	[50]
Ni–Co–Cu oxide nanorods (10–40 nm)	−0.1 ÷ 0.65 vs. Hg/HgO	3	6.54 F/cm^2^	the device retains about 40% after 2000 cycles at 2 A/g	[25]
Cu nanoparticles/PCNFs	0 ÷ 1	1	333.5	retains about 95.8% after 10,000 cycles at 3 A/g	[51]
Si/MnO_2_ nanoneedle (20–40 nm)	−0.2 ÷ 0.8 vs. Ag/AgCl	1	240.1	retains about 85.2% after 2000 cycles at 1 A/g	[52]
PANI-Si nanoparticles (1–2.8 nm)	−0.2 ÷ 1 vs. Ag/AgCl	5 mA/cm^2^	470	retains 78% after 1500 cycles	[53]
CeCu_2_Si_2_ rough micro pebbles	−0.4 ÷ 0.7 vs. SCE	1	278	retains about 98% after 20,000 cycles at 10 A/g	This work

## Data Availability

No new data were created or analyzed in this study. Data sharing is not applicable to this article.
